# 1270. Impact of a Multidisciplinary Antimicrobial Stewardship Program (ASP) on Antibiotic Utilization and Clinical Outcomes at Tertiary Hospital in Saudi Arabia: A Quasi-experimental Study

**DOI:** 10.1093/ofid/ofad500.1110

**Published:** 2023-11-27

**Authors:** Khadijah Sarkhi, Abrar K Thabit, Khalid Eljaaly, Reham Kaki, Rahaf Bahamdan, Sultan A Alghamdi, Mohammed O Baharith

**Affiliations:** 1) Pharmaceutical Care Services, King Abdulaziz University Hospital, Jeddah, Saudi Arabia. 2) Pharmaceutical Care Services, King Fahad Armed Forces Hospital, Jeddah, Saudi Arabia, Jeddah, Makkah, Saudi Arabia; King Abdulaziz University, Jeddah, Makkah, Saudi Arabia; King Abdulaziz University, Saudi Arabia, Jeddah, Saudi Arabia; King Abdulaziz university, Department of Infectious Disease and Department of Infection Control and Environmental Health , Jeddah, Saudi Arabia, Jeddah, Makkah, Saudi Arabia; King Abdulaziz University Hospital, Jeddah, Makkah, Saudi Arabia; King Abdulaziz University, Jeddah, Makkah, Saudi Arabia; King Abdulaziz University, Jeddah, Makkah, Saudi Arabia

## Abstract

**Background:**

The concept of antimicrobial stewardship has been introduced for over a decade in response to the rapid global emergence and spread of antimicrobial resistance. To measure the impact of ASP interventions, several ASP metrics were developed. Very limited studies from Saudi Arabia have evaluated the impact of ASP interventions on antibiotic use and clinical outcomes of non-ICU patients. Therefore, we aimed to assess the impact of this ASP intervention on various ASP metrics by comparing the data of the post-intervention phase to that of the pre-intervention phase.

**Methods:**

A quasi-experimental study was performed on patients admitted to non-ICU wards for whom a restricted antibiotic was ordered. Prospective audit and feedback (PAF) were already in place; however, on September 1st, 2020, a bundle of changes was introduced to improve the intervention making it a multifaceted intervention. These included the involvement of an infectious diseases clinical pharmacist, review of orders by attending (consultant) infectious diseases physicians, utilization of electronic resources instead of paperwork, and improvement of documentation. The primary outcomes were antibiotic utilization measures as defined daily dose per 100-patient days (DDD/100PD) and days of therapy per 100-patient days (DOT/100PD). Secondary outcomes were clinical outcomes of patients.

**Results:**

A total of 402 episodes were evaluated for 167 and 188 unique patients in the pre- and post-intervention phases, respectively. DDD/100PD and DOT/100PD in the pre phase vs the post phase were 1.75 vs. 2.54 and 16.13 vs. 44.93, respectively (*P* < 0.001 for both comparisons). Antibiotic de-escalation and clinical cure rates were significantly higher in the post phase than in the pre phase (62% vs. 40.6% and 83.5% vs. 65.8%; *P* < 0.001 for both comparisons). In-hospital mortality and 30-day readmission rates were lower in the post phase than in the pre phase (13% vs. 20.8%; *P* = 0.037 and 20.5% vs. 33.8%; *P* = 0.003, respectively). Length of stay in both hospital and ICU were shorter in the post phase (14 vs. 22 days and 3 vs. 9 days, respectively; *P* < 0.001 for both comparisons).

**Conclusion:**

Our study demonstrates that the ASP intervention was associated with significant improvement in antibiotic utilization and patients’ clinical outcomes.

**Disclosures:**

**Khalid Eljaaly, PharmD, MS, BCPS, BCIDP, FCCP**, Reckitt Benckiser Healthcare International Ltd, UK: Member of the Global Respiratory Infection Partnership, supported by unrestricted educational grant from Reckitt Benckiser Healthcare Ltd

